# Comparison of Barriers to Cessation among Arab American Smokers of Cigarettes and Waterpipe

**DOI:** 10.3390/ijerph110909522

**Published:** 2014-09-15

**Authors:** Linda Haddad, Omar El-Shahawy, Roula Ghadban

**Affiliations:** 1 College of Nursing, University of Florida, Gainesville, FL 32604, USA; 2 Department of Social and Behavioral Health, School of Medicine, Virginia Commonwealth University, Richmond, VA 23219, USA; E-Mail: elshahawyo@vcu.edu; 3 School of Nursing, Virginia Commonwealth University, Richmond, VA 23219, USA; E-Mail: ghadbanr@vcu.edu

**Keywords:** dual smokers, waterpipe smokers, Arab American, barriers to cessation

## Abstract

This cross-sectional study examined the differences in barriers to cessation and reasons for quitting smoking among dual smokers of cigarettes and waterpipe tobacco, exclusive cigarette smokers and exclusive waterpipe smokers. Participants were Arab American adults residing in Richmond, Virginia, who were recruited from Middle Eastern grocery stores, restaurants/lounges and faith and charity organizations. The study yielded several key findings: (1) Exclusive cigarette and waterpipe smokers had similar mean barriers to quitting and were more concerned about their health than dual smokers. (F(2, 150) = 5.594, *p* = 0.0045). This implies that barriers to smoking and health concerns could be a function of the individual who smokes rather than the modality of smoking itself. (2) Exclusive cigarette or waterpipe smokers and dual smokers may have different reasons for quitting, since they have different reasons for smoking. The proportion of smokers who endorsed smoking as a messy habit as the reason among exclusive cigarette smokers was 0.37, whereas the proportion among exclusive waterpipe smokers was 0.04 and among dual smokers 0.39. The difference in proportions is significant, χ^2^ (df = 2, N = 154) = 13.17, *p* = 0.0014. In summary, this study supports the need to further investigate dual cigarette and waterpipe smokers, as the study results indicate greater barriers to smoking cessation in this group. Recognition and understanding of these barriers among dual tobacco users would be important for any future tobacco intervention among waterpipe smokers.

## 1. Introduction

Waterpipe tobacco smoking (WTS) is growing steadily on a global level [1,2,3], including in the United States (U.S.) [4,5,6]. Nicotine delivery via waterpipe, unlike cigarette smoking, is variable [7,8], due to the differences in the length, frequency and depth of inhalation [9,10]. Both cigarette smoking and WTS lead to dependence [9,11,12], but WTS has a strong social aspect that makes it particularly appealing and addicting to users [13,14]. The prevalence of cigarette smoking in the U.S. is currently 18.1% [11,15], and waterpipe smoking is considered to be 8.8% [16]. In addition, there are still more dual users of cigarettes and waterpipes than there are exclusive waterpipe smokers [15,16,17,18]. A study by Cobb *et al.* (2005) showed that there are more dual users of cigarettes and waterpipes than there are exclusive waterpipe smokers [5]. Nevertheless, dual use appears to be associated with more reported nicotine dependence and barriers to cessation than exclusive cigarette use [15]. Other studies have shown that some cigarette smokers report switching to WTS as an approach to smoking cessation [18], which is fueled by a myth that quitting waterpipe smoking is much easier than quitting cigarette smoking [19].

Despite the presence of multiple barriers to quitting, there are a number of factors that could trigger a quit attempt. Tobacco use cessation counseling by a physician is perceived by tobacco users as a strong prompt to quit smoking [20,21,22,23] and has been repeatedly shown to significantly increase quitting rates [24,25,26]. Others reasons for quitting include family members’ advice and smoking being a messy habit, among others [27]. In general, quitting smoking is a difficult task, and no studies have compared barriers and reasons for those barriers between cigarette, waterpipe and dual users. In practice, as the number of dual users becomes greater than exclusive waterpipe users and much less than cigarette users, there is a need to explore the differences in barriers to cessation between these three groups to inform cessation activities.

In summary, there are limited studies that evaluate potential differences in barriers to cessation and reasons for quitting among dual smokers, exclusive cigarette smokers and exclusive waterpipe smokers. Thus, the purpose of this study was to examine the barriers to cessation among dual users to: (1) increase our understanding of the barriers to cessation among dual users of cigarettes and waterpipes; and (2) gain perspective regarding the similarities and differences of either dual or exclusive smokers’ barriers to cessation and quitting behaviors. We have focused on Arab Americans since the prevalence of waterpipe smoking is thought to be higher among them, due to cultural reasons, and this would enable easier recruitment of waterpipe users. For example, a study comparing waterpipe and cigarettes use among Arab American and non-Arab-American youth found the prevalence of waterpipe smoking to be 17% *vs.* 11% among Arab Americans [28].

## 2. Methods

### 2.1. Participants and Procedure

Participants were recruited from the Richmond, Virginia, metropolitan area through a convenience sampling technique. Details regarding the recruitment procedures and the overall sample are published elsewhere [15]. A power analysis was conducted to determine the required study sample size. The minimum sample size required for a two-sided significance level of 95%, a power of 80%, with prevalence of tobacco use among Arab American estimated to be 35% [28,29], was estimated to be a total of 150 subjects. In summary, participants were sought through advertising in social media and in places where Arab-Americans are usually present, like cultural centers, Middle-Eastern grocery stores, *etc.* A contact number was provided for them to call if they were interested in participation. Only current smokers were included, as the focus of this study was on contrasting barriers to cessation between exclusive cigarette smokers, exclusive waterpipe smokers and dual smokers. The total final study sample that was analyzed consisted of 154 smokers.

### 2.2. Measurements

Specific to our present analysis, we focused on three types of tobacco smokers: (1) exclusive cigarette smokers, defined as participants reporting exclusive cigarette smoking with no WTS use in the past 30 days; (2) dual users, defined as participants reporting both cigarette and waterpipe use in the past 30 days; and (3) exclusive waterpipe users, defined as participants reporting exclusive waterpipe use in the past 30 days. Participants from the three groups completed the study questionnaire, which included the following instruments: 

i. Demographic and Cultural Information Questionnaire: A 21-item instrument that was used to obtain demographic and cultural information, as well as other relevant information for such an immigrant population, such as country of origin and language spoken at home [30]. Relevant results pertaining to acculturation effect on the sample have been published elsewhere [15].

ii. Tobacco Use and Smoking History Questionnaire: A 32-item questionnaire that pertains to smoking history habits, past quit attempts, attitudes, beliefs towards tobacco use and desire to quit. This questionnaire was previously used by Haddad and Petro (2006) and showed high validity and reliability [30]. For the purpose of this study, we focused on two questions regarding personal health and perceptions of smoking harm, as well as the reasons for quitting. The latter was addressed by the following question: “Which of the following seem to be your reasons for wanting to quit or cut down your smoking? (Check as many as apply)” with the following 11 response categories: “the cost of smoking, to improve my sense of taste or smell, the messiness or dirtiness of the habit, the effect of smoking on my health, having my doctor tell me to stop or cut down, scientific reports on the dangers of smoking, being a bad example on the dangers of smoking, having a spouse or family members who want me to stop or cut down, not really enjoying smoking, other and I don’t want to quit or cut down”.

iii. Barriers to Cessation Scale: A 19-item questionnaire that is comprised of three subscales in addition to a “gaining weight” item [31]. The three subscales are: the Addiction Barriers subscale (8 items), the External Barriers subscale (7 items) and the Internal Barriers subscale (3 items). The scale is composed of 4 categories starting with -1- “always a barrier” and end with -4- “not at all”. Nineteen is the lowest total score, and 74 is the highest total score; participants with barriers scores of 60 and above are classified as having low barriers and as ready for smoking cessation [31].

### 2.3. Analysis

We used the JMP version 10 statistical package to analyze the study data. Relevant statistics were calculated for the entire sample and then stratified by smoking status (e.g., exclusive waterpipe or cigarette smokers and dual smokers). First, we described the sample for each group. Then, we used equal variance ANOVA to examine the mean differences between the three groups regarding barriers to cessation scores, concerns of smoking harm, which was assessed with the question, “Do you think smoking is harmful to health?” (strongly agree, mildly agree, mildly disagree, strongly disagree, no opinion/don’t know), and concern regarding the effect of smoking on health, which was assessed with the question, “Are you concerned about the harmful effect smoking may have on your health?” (very concerned, fairly concerned, slightly concerned, not concerned). Concerns about health and perception of smoking harm were coded as ordinal level variables with four levels each. If the overall F-test was found to be statistically significant, *post hoc* pairwise comparisons of the means were performed using Tukey’s HSD method. We also used the chi square test to evaluate the differences between the three groups regarding different reasons for quitting. Data were examined regarding distributions and trends with the level of significance set to *p* < 0.05.

## 3. Results

### 3.1. Sample Characteristics

Our sample was comprised of 154 smokers. Among those, 18.2% (*n* = 28) were dual users (*i.e.*, cigarettes and waterpipe) and 15% (*n* = 22) were exclusive waterpipe smokers. Within the total sample, males comprised 67.5% (*n* = 104) with a mean age of 28.1 years (SD = ±10.1). Exclusive cigarette smokers were mostly males (70%), with a mean age of 28 years (SD = ±10.3), and the mean number of daily cigarettes smoked was 8.9 (SD = ±6.1). Dual smokers were mostly males (77%), with a mean age of 27.8 years (SD = ±10.6); the mean number of daily cigarettes smoked was 12.9 (SD = ±7.9), and the mean number of daily waterpipe sessions was 1.1 (SD = ±0.33). Exclusive waterpipe smokers were mostly males, comprising 52% (*n* = 12), with a mean age of 28.8 years (SD = ±9.3), and the mean number of daily waterpipe sessions was 1.2 (SD = ±0.6).

### 3.2. Reasons for Quitting

See Table 1 for data pertaining to the reasons for quitting in the current sample (*n* = 154). As seen in Table 1, contingency analysis was performed to determine the differences between groups regarding the proportions of the ten possible listed reasons for quitting tobacco. There were significant differences between the proportions of the three groups for two of the reasons. The proportion of smokers who endorsed smoking as a messy habit as the reason among exclusive cigarette smokers was 0.37, whereas the proportion among exclusive waterpipe smokers was 0.04 and among dual smokers 0.39. The difference in proportions is significant, χ^2^ (df = 2, N = 154) = 13.17, *p* = 0.0014. The proportion of smokers who endorsed family members’ advice as the reason for quitting among exclusive cigarette smokers was 0.28, whereas the proportion among exclusive waterpipe smokers was 0.04 and among dual smokers 0.29. The difference in proportions is significant, χ^2^ (df = 2, N = 154) = 9.55, *p* = 0.0084.

**Table 1:** Reasons for quitting by smoker type.

Reason to Quit	Dual Smokers	Cigarette Only	Waterpipe Only
	N = 28 (%)	N = 104 (%)	N = 22 (%)
Cost of smoking	68%	47%	4%
Improve my sense of taste or smell	32%	29%	8.7%
Messiness or dirtiness of the habit	39%	38%	4%
The effect of smoking on my health	10.7%	27%	4%
Having my doctor tell me to stop or cut down	10.7%	16.5%	0%
Scientific reports on the dangers of smoking	35%	17.4%	0%
Being a bad example on the dangers of smoking	14.3%	29%	0%
Having spouse or family members who want me to stop or cut down	46.4%	28%	8%
Not really enjoying smoking	7%	11.6%	8%
I don’t want to quit or cut down	7%	10%	4%

Dual smokers had higher percentage rates than exclusive smokers for endorsing the following five reasons for quitting: cost of smoking, improving sense of taste or smell, messiness of smoking, dangers of smoking and family members’ support to quit. On the other hand, participants who smoked cigarettes exclusively had higher percentages of the remaining reasons: the effect of smoking on health, doctors’ advice, not enjoying smoking and not wanting to quit. Exclusive waterpipe smokers had the lowest percentage for all 11 reasons mentioned earlier (Table 1).

### 3.3. Barriers to Cessation Differences between the Three Smoker Groups

Overall Barriers: The scores for the total sample ranged from two to 74. The mean scores and standard deviations of the scale are available in Table 2. An ANOVA test indicated that the group means were significantly different (F(2, 150) = 5.594, *p* = 0.0045). Using Tukey’s HSD, it was determined that dual smokers had a significantly higher mean overall score than that of the other smoker groups, with no other significant differences found. Table 2 also summarizes the estimated differences.

Addictive Barriers: The scores for the total sample ranged from zero to 33. A summary of the mean overall barriers by group is shown in Table 2. An ANOVA test indicated that the group means were significantly different (F(2, 151) = 15.21, *p* < 0.0001). Using Tukey’s HSD, it was determined that dual smokers had a significantly higher mean score than exclusive cigarette smokers and that exclusive cigarette smokers had a significantly higher mean score than exclusive waterpipe smokers. No other significant differences were found. Table 2 also summarizes the estimated differences.

External Barriers: The scores for the total sample ranged from zero to 28. A summary of the mean overall barriers by group is shown in Table 2. An ANOVA test indicated that the group means were significantly different (F(2, 151) = 3.465, *p* = 0.0338). Using Tukey’s HSD, it was determined that dual smokers had a significantly higher mean score than exclusive cigarette smokers. No other significant differences were found. Table 2 also summarizes the estimated differences.

Internal Barriers: The scores for the total sample ranged from zero to 15. A summary of the mean overall barriers by group is shown in Table 2. An ANOVA test indicated that the group means were not significantly different (F(2, 151) = 2.9508, *p* = 0.0553).

**Table 2:** Summary of barriers and concern about health scores.

Outcome Variables	Dual	Cigarette	Waterpipe
	N = 28	N = 104	N = 22
	M (SD)	M (SD)	M (SD)
	95% CI	95% CI	95% CI
Overall Smoking Cessation Scale	45 (9)	38 (13)	33 (11)
	41.62, 48.81	35.91, 41.02	28.63, 39.10
Addiction Barrier	16 (4)	13 (6)	6 (7)
	14.31, 18.12	11.77, 14.47	2.80, 9.37
Internal Barrier	8 (2)	7 (3)	5 (3)
	7.31, 9.48	6.40, 7.89	4.28, 7.55
External Barrier	19 (4)	16 (6)	18 (6)
	17.58, 21.27	15.07, 17.49	15.37, 21.32
Health Concern Perception	1 (1)	1 (0.9)	1 (0.8)
	0.75, 1.5	1.67, 2.05	1.58, 2.33

### 3.4. Health Perception and Tobacco User Group

Perceptions of smoking harm did not differ significantly between the three groups, with most of the participants strongly or mildly agreeing that smoking is harmful to health. However, the concerns of smoking harm on the smoker differed significantly between the three groups. A summary of the mean concern of smoking harm by group is shown in Table 2. An ANOVA test indicated that the group means were significantly different (F(2, 151) = 6.6985, *p* = 0.0016). Using Tukey’s HSD, it was determined that dual smokers had a significantly lower overall mean score than each of the other smoker groups. No other significant differences were found.

## 4. Discussion

Dual smokers appeared to have more barriers to cessation than either of the other two groups: exclusive cigarette and exclusive waterpipe smokers. Despite the fact that all three groups acknowledged that smoking is harmful to health in general, dual smokers had significantly less concern about their own health compared to either of the two remaining groups. Moreover, two reasons for quitting—the perception of smoking being a messy and/or dirty habit and family support—differed significantly between the three groups. Exclusive waterpipe smokers scored significantly lower on these measures than members of the other two groups.

These findings highlight a concern for smoking cessation activities. Dual smokers appeared to have more barriers and fewer concerns for the harm of smoking than exclusive smokers of either cigarettes or waterpipes. For example, exclusive waterpipe smokers had significantly lower scores on the reasons for quitting regarding their perception of smoking as a messy and/or dirty habit and family support to quit smoking. This finding may have been due to the nature of smoking waterpipes; it usually occurs in hookah bars away from the family, where it does not create “a mess”. These findings are in line with interpretations of Arab-American smokers perceptions and attitudes found through focus groups in a study by Kuiwicki *et al.* (2003) [29]. Participants of that study exhibited perceptions of safety of waterpipe smoking relative to cigarettes smoking and how waterpipe smoking usually occurs outside the home environment in the hookah bars where they go and “hang out with the guys”. The number of cafes for waterpipe smoking has tremendously increased across the U.S., especially in proximity of colleges and universities [32]. With respect to other barriers, the findings raised a poignant question: do dual smokers have more barriers to smoking cessation because they use more tobacco products or because they are simply more addicted to nicotine? This cause/effect relationship, which was beyond the scope of our study, represents an important avenue for future research. Simultaneously, dual smokers may have more barriers to cessation, but it appears that a higher proportion want to quit or cut down compared to exclusive waterpipe smokers. This might indicate that the addictiveness to nicotine may be imposing a significant barrier that prevents those dual smokers from taking an action or the permissiveness of dual use of tobacco products itself makes them smoke in more places, which limits the effect of smoking bans on those smokers. Furthermore, some explanation could be related to our first published part of the study, where we found that more acculturation implied less nicotine dependence among subjects of the study [15].

Waterpipe smoking, with its pleasant aroma and profound social context, is a pleasant experience in a social setting [13]. While some waterpipe smokers use only waterpipes as a source of nicotine, most waterpipe smokers use it as a supplement to cigarettes [17]. In fact, our results showed that dual smokers smoked more cigarettes than exclusive cigarette smokers, but because the literature lacks studies in this area, we were unable to validate this finding. In addition, our data show that dual smokers have significantly more barriers and fewer health concerns than both exclusive smoker groups. This poses a significant challenge to the belief that exclusive cigarette smokers tend to switch to waterpipe smoking in an attempt to quit smoking. Cigarette smokers may initially intend to quit when they begin smoking waterpipes, but our results indicate that dual smoking may have a synergistic or an enabling effect; indeed, exclusive cigarette smokers may become more addicted when they begin smoking waterpipes in addition to cigarettes. Thus, contrary to this popular assumption, it is possible that highly addicted smokers begin to smoke waterpipes because they are seeking tobacco in other forms and not because they intend to quit.

Exclusive cigarette and waterpipe smokers had similar mean barriers to quitting and were more concerned about their health than dual smokers. This implies that barriers to smoking and health concerns could be a function of the individual who smokes rather than the modality of smoking itself. It is well known that cigarette and waterpipe smoking is harmful, but only dual smokers express significantly more barriers to quitting and fewer personal health concerns. Since we cannot ascertain whether dual smokers are more addicted and have more barriers prior to experiencing multi-tobacco use or whether they eventually just become more dependent on nicotine, future longitudinal studies are needed to follow tobacco users and monitor their patterns of use to reveal this interaction and understand the effects of using multiple tobacco products.

This study suggests a need for future research to focus on dual tobacco use, as it could become more prevalent and would pose specific challenges to cessation efforts. Nevertheless, our study is subject to a number of limitations. First, the study used a convenience sample. Unfortunately, this type of sampling in surveys is vulnerable to potential response biases that might affect the result. This limits the generalizability of the study results. Second, these data rely on self-reporting, which is subject to recall bias, as well as any social stigma associated with tobacco use among Arab Americans. The social unacceptability of smoking in the U.S. is very evident and has helped decrease smoking rates [33]. However, this would most likely not affect our results, since participants were reassured of the confidentiality of their responses. Nonetheless, simultaneous use of cigarettes and waterpipes among Arab American adults is more common than reported in some other populations [15] and has an epidemiology that is distinct from either exclusive use of cigarettes or exclusive use of waterpipe tobacco. Knowledge of barriers to cessation and reasons to quit would contribute to the currently limited research on dual tobacco use and the development of future tobacco interventions for Arab American adults.
